# Exploring Structure-Sensitive Factors Relevant to Cryogenic Laser Operation in Oxide Crystals Doped with Er^3+^ Ions

**DOI:** 10.3390/ma16052095

**Published:** 2023-03-03

**Authors:** Witold Ryba-Romanowski, Radoslaw Lisiecki, Jaroslaw Komar, Boguslaw Macalik, Marek Berkowski

**Affiliations:** 1 Institute of Low Temperature and Structure Research, Polish Academy of Sciences, ul. Okólna 2, 50-422 Wrocław, Poland; 2 Institute of Physics, Polish Academy of Sciences, Al. Lotników 32/46, 02-668 Warsaw, Poland

**Keywords:** cryogenic lasers, Er-doped crystals, optical spectroscopy, luminescence

## Abstract

Crystals of Gd_3_Al_2.5_Ga_2.5_O_12_:Er^3+^, (Lu_0.3_Gd_0.7_)_2_SiO_5_:Er^3+^ and LiNbO_3_:Er^3+^ compounds differing in origin and the nature of their inherent structural disorder were crystalized. Optical absorption and luminescence spectra for transitions between the ^4^I_15/2_ and the ^4^I_13/2_ multiplets of Er^3+^ ions for the crystal samples were recorded versus temperatures in the region of 80–300 K. Gathered data were analyzed thoroughly providing the in-depth knowledge of the effects of temperature on intensities, wavelengths and bandwidths of Er^3+^ transitions. The information acquired together with the knowledge of significant structural dissimilarities of the host crystals chosen made it possible to propose an interpretation of the impact of a structural disorder in Er^3+^-doped crystals on their spectroscopic properties, and to determine their lasing ability at cryogenic temperatures upon resonant (in-band) optical pumping.

## 1. Introduction

Numerous papers published in the last decade have been devoted to the lasing of rare earth-doped crystals at cryogenic temperatures. The most thoroughly studied were cryogenic lasers based on YAG crystals, e.g., YAG:Ho [1,2], YAG:Tm [3,4], YAG:Er [5], and YAG:Yb [6,7], although other systems have also been studied, e.g., LuVO_4_:Tm,Ho [8], LuVO_4_:Er [9], GdVO_4_:Er [10,11], YVO_4_:Er [12], YLF:Yb [13,14], YLF:Ho [15]. It follows from these results that the laser performance of rare earth-doped crystals at cryogenic temperatures is better, in that their laser efficiency is higher and their laser thresholds are reduced. In addition, cryogenic cooling combined with a resonant (on-line) optical pumping is able to markedly reduce heat generation, thereby preventing the creation of adverse thermal gradients that induce stresses even at high lasing powers. 

The papers cited above deal with crystal hosts with an ordered structure. Although the group of rare earth-doped crystals hosts with significant inherent structural disorder, termed disordered crystals, is wide, knowledge on their laser potential at cryogenic temperatures is rather poor. As a matter of fact, interest has mainly been directed towards Gd_3_Ga_5_O_12_-Gd_3_Al_5_O_12_ (GAGG) solid solutions doped with rare earth ions. Cryogenic conditions were used to improve the efficiency of a diode-pumped tunable Tm, Ho:GGAG laser [16]. The effect of temperature (in the range 78–400 K) on the spectroscopic and laser features of Yb^3+^ ions in the garnet Gd_3_Ga_x_Al_5-x_O_12_ (Yb:GGAG) have been documented [17]. Additionally, some spectroscopic parameters and the laser performance of Er-doped Gd_3_Ga_3_Al_2_O_12_ crystal (Er:GGAG) were investigated at a temperature between 80 and 340 K [18]. Structural disorder gives rise to distortion of the crystal field acting on incorporated luminescent ions. As a consequence, their spectral lines show inhomogeneous line broadening. This feature is advantageous because it improves the overlap between the absorption bands of a medium and the emission lines of laser diodes employed for optical pumping. Additionally, the inhomogeneous line broadening of spontaneous emission of incorporated luminescent ions may endow rare-earth doped crystals with an ability to emit ultrashort laser pulses.

In the present study, we focus the attention on crystal hosts characterized by a certain structural disorder, which show an ability to induce strong spectral broadening of absorption and luminescence lines of incorporated rare earth ions. The Gd_3_(Al_0.5_Ga_0.5_)_5_O_12_ (GAGG) and (Lu_0.3_Gd_0.7_)_2_SiO_5_ (LGSO) solid solutions and a congruent LiNbO_3_ (LNO) were chosen because their crystallization by the Czochralski method has been mastered already, and their structure and properties are well documented [19,20,21,22,23,24,25,26,27,28,29,30,31,32,33,34,35].

The Gd_3_Ga_5_O_12_-Gd_3_Al_5_O_12_ (GAGG) solid solution is denoted as substituted or mixed garnets. There is an abundance of literature regarding the fundamental properties of GAGG [19,20,21,22]. In a general chemical formula of garnets {A3}[B2](C3)O12, the symbols {A3},[B2] and (C3) denote cations in dodecahedral sites with a coordination number equal to 12, cations in octahedral sites with coordination number equal to 6 and cations located in tetrahedral sites with coordination number equal to 4, respectively, and O denotes oxygen. A general formula for GAGG reads {RE3}[B2}(B3)O12, where RE denotes rare earth ion and B is Al or Ga. Accordingly, in the GAGG structure, the Al or Ga ions occupy octahedral and tetrahedral sites, and incorporated Er ions substitute Gd ions.

The Lu_2_SiO_5_–Gd_2_SiO_5_ (LGSO) solid solution was invented for technical reasons [23,24,25,26,27,28,29]. The main reason for substituting Lu by Gd in a well-known Lu_2_SiO_5_:Ce^3+^ scintillating material is to reduce its melting temperature. Another advantage is that the price of its fabrication may be lowered by replacing the expensive Lu_2_O_3_ with a much cheaper Gd_2_O_3_. It has been demonstrated [24,29] that almost 85% of Lu may be replaced without changing the structure of Lu_2_SiO_5_ from C2/c (LSO) to the P21/c of Gd_2_SiO_5_ (GSO). This change is therefore advantageous, offering a reduced melting point and fabrication cost as well as homogeneous distribution of dopant in a matrix.

LiNbO_3_ crystals have been known for a long time; however, some their features are not clear. Reported band-gap values range from 3.28 eV [30] to 4.3 eV [31]. The distribution of cations among available sites in congruent LiNbO_3_ crystals has been interpreted in the framework of the Nb site vacancy model [32] or the Nb split models [33]. The same problem concerns the location of incorporated luminescent rare earth ions. It has been found that Eu^3+^ ions substitute the Li^+^ and Nb^5+^ ions [34]. In another study, it has been observed that Pr^3+^, Ho^3+^, Yb^3+^ ions in LiNbO_3_ substitute Li^+^ ions, which are slightly shifted from the regular positions [35].

## 2. Materials and Methods

**Figure 1:**
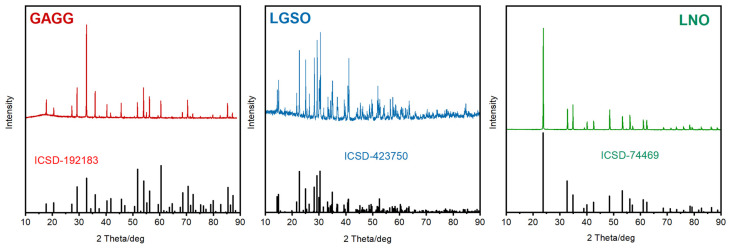
XRD patterns of GAGG:Er, LGSO:Er and LNO:Er single crystals.

Crystals of Gd_3_Al_2.5_Ga_2.5_O_12_ (GAGG) containing 0.5 at% Er^3+^ (6.25 × 10^19^ ions/cm^3^) and (Lu_0.3_Gd_0.7_)_2_SiO_5_ (LGSO) containing 0.5 at% of Er^3+^ (9.05 × 10^19^ ions/cm^3^) were fabricated by the Czochralski method from iridium crucibles. More detailed information regarding the growth procedure and fundamental structural and spectroscopic features of these compounds are given in [36,37], respectively. The structure of all studied crystals has been verified, and as a result, the XRD patterns and adequate data taken from ICDS base can be seen in Figure 1. Furthermore, the relevant physicochemical properties of the studied hosts are listed in Table 1. Stoichiometry of the host crystals was checked based on the results of X-ray measurement, and the concentration of admixtures was determined by ICP-EM examination. A crystal of LiNbO_3_ (LNO) doped with 1.98% Er^3+^ (5.64 × 10^19^ ions/cm^3^) was grown from a lithium-deficient (Li/Nb = 0.94) melt from a platinum crucible [38]. Anisotropy of LGSO was determined referring to the crystallographic b axis and the directions of indicatrices X1 and X2 [39]. An anisotropic, uniaxial LNO:Er sample was oriented based on a crystallographic method. Absorption spectra were measured at several temperatures within the 6 K–300 K with a Varian Model 5E UV–VIS–NIR spectrophotometer. The spectral bandwidth of the instrument was set to 0.1 nm. Emission spectra as function of temperature in the region 6 K–300 K were acquired using a DongWoo Optron DM 711 grating monochromator with 750 mm focal length, and an InGaAs detector. The crystals samples were placed into an Oxford Instr. model CF 1204 continuous flow liquid helium cryostat equipped with a temperature controller. An infrared diode laser CNI MDL-III-803R emitting at 803 nm was used for the excitation. Acquired emission spectra were corrected for the spectral response of the experimental setup.

## 3. Treatment of Experimental Data

A dual-beam absorption spectrophotometer provides the absorption intensity of a measured sample in units of dimensionless absorbance *A* = *log*_10_(*I*_0_/*I_t_*), where *I*_0_ is the incident beam intensity and *I_t_* is the transmitted beam intensity. Following the Beer–Lambert law, the transmitted intensity fraction *T* = *I_t_*/*I*_0_ = *exp*(−*αd*), where *α* is absorption coefficient and *d* is sample thickness. Accordingly, *A* = (*ln*10)*αd* and *α*~*A*/0.43*d*. In this way, an absorption spectrum can be conveniently expressed as a plot of *α* versus wavelength *λ*. To compare the absorption ability of samples differing in chemical composition and/or density of absorbing ions, an absorption spectrum is expressed in cross-section units (σ*_a._*) according to the following relation:

(1) $$\sigma_{a.}=\frac{\alpha }{N}$$

where *α* denotes the absorption coefficient and *N* denotes the concentration density of the Er^3+^ ions.

In principle, a spectrum of *σ_a._*(*λ*) for a transition of trivalent rare earth ions from the ground state to a first higher-energy-excited multiplet is related to the spectrum of the reverse (luminescent) transition [40]:

(2) $$\sigma_{e}^{p}\left(\lambda \right)=\sigma_{a}^{p}\left(\lambda \right)\frac{Z_{f}}{Z_{i}}\exp\left[\left(E_{zl}-hv\right)/kT\right]$$

where *Z_f_*, and *Z_i_* are partition functions of distribution of the ground state and excited multiplet, respectively. They are defined as [40]

(3) $$Z_{f}=\sum_{k}g_{k}e^{-E_{k}/kT},Z_{i}=\sum_{j}g_{j}e^{-\left(E_{j}-E_{zl}\right)/kT}$$

where *E_k_* i *E_j_* are energies of crystal field levels and *g_k_* i *g_j_* stand for their degeneracies. *E_zl_* denotes the energy difference between the lowest-energy crystal field levels of the multiplets involved.

Commonly, the Füchtbauer–Ladenburg (FL) method is used to determine the emission cross-section from luminescence spectra as follows [41]:

(4) $$\sigma_{e.}\left(\lambda \right)=\frac{\beta }{8\pi n^{2}c\tau }\frac{\lambda^{5}I\left(\lambda \right)}{\int \lambda I\left(\lambda \right)d\lambda }$$

where *I(λ)/∫λI(λ)dλ* is a normalized line shape function of the experimental emission spectrum *I(λ),* luminescence branching ratio *β = 1* for the ^4^I_13/2_→^4^I_15/2_ Er^3+^ transition, *τ* is the ^4^I_13/2_ radiative lifetime, *n* is the refractive index and *c (m/s)* is the velocity of light. To obtain spectra from Equation (4), the radiative lifetime values *τ* should be known. The relaxation rate of the ^4^I_13/2_-excited state of Er^3+^ is a sum of the electric dipole transition rate *A(ED)* and of the magnetic transition rate *A(MD).* The *A(ED)* can be evaluated by employing the following relation [42]:

(5) $$A\left(ED\right)=\frac{64\pi^{4}e^{2}}{3h\left(2J+1\right)\lambda^{3}}\frac{n{\left(n^{2}+2\right)}^{2}}{9}\sum_{t=2,4,6}\Omega_{t}{\left|\langle l^{N}\psi J||U^{\left(t\right)}||l^{N}\psi^{\prime }J^{\prime }\rangle \right|}^{2}$$

where |<Ut>| are matrix elements of unit tensor operator for the ^4^I_13/2_→^4^I_15/2_ transition, and *Ω_t_* are phenomenological parameters determined by the Judd–Ofelt (J-O) treatment of absorption spectra [42]. The contribution of the *A(MD)* is rather small. It can be calculated using relations given elsewhere [43]. Alternatively, the value of the *A(ED)* may be found based on the relation [43] between the oscillator strength *P(ED)*

(6) $$P\left(ED\right)=4.32\cdot 10^{-9}\cdot \int \frac{A}{c}d\nu$$

where A is absorbance and c is the Er^3+^ concentration and A(ED) is as follows:

(7) $$A\left(ED\right)=\frac{8\pi^{2}e^{2}n^{2}}{mc\lambda^{2}}\times \frac{2J^{\prime }+1}{2J+1}\times P\left(ED\right)$$

where *n* is the refractive index, the values of *J′* and *J* are 13/2 and 15/2, respectively, and *λ* denotes emission wavelength. Symbols *c*,* m* and *e* denote the speed of light, electron mass and electron charge, respectively.

The inverse of a sum *A*(*ED*) + *A*(*MD*) is the ^4^I_13/2_ radiative lifetime *τ*. The reliability of the values calculated depends on the inherent incertitude of the approaches given above. We compare results of the calculation employing Equations (5) and (7). Actual emission cross-section values for the ^4^I_13/2_→^4^I_15/2_ transition will be smaller due to the adverse effect of self-absorption, hence the gain coefficient G for a predicted laser operation is [44]

(8) $$G\left(\lambda \right)=N\left[K\sigma_{e.}\left(\lambda \right)-\left(1-K\right)\sigma_{a.}\left(\lambda \right)\right]$$

where *N* is the Er^3+^ ion concentration and *K* denotes a population inversion parameter defined as the ratio of erbium ion density in the excited state to the overall erbium ion density in the crystal.

The Er^3+^ effective emission wavelength *λ_eff._* as a function of temperature for crystals studied was calculated utilizing the equation [45]:

(9) $$\lambda_{eff.}=\frac{\int \lambda I_{f}\left(\lambda \right)d\lambda }{\int I_{f}\left(\lambda \right)d\lambda }$$

where *I_f_ (λ)* = $cont.\cdot \frac{\sigma_{e.}\left(\lambda \right)}{\lambda^{5}}$.

## 4. Results and Discussion

Several factors are responsible for the effects of temperature on absorption and emission spectra. Thermal line broadening, a mechanism consisting of Raman scattering of phonons by an ion in an excited state, and thermal line shift, which determines the change of transition energy due to temperature-induced displacement of levels involved in the transitions, are factors that accompany the temperature-dependent populations of initial and terminal levels.

In the 4f^11^ configuration of Er^3+^ ions, the lowest energy multiplets are the ^4^I_15/2_ ground state and the ^4^I_13/2_ first excited state. They are separated by about 6500 cm^−1^. When Er^3+^ ions are incorporated in a crystal host, the crystal field splits the ground multiplet into eight crystal field levels, and the excited multiplet into seven crystal field levels. In addition, the crystal structure of numerous hosts presents a certain disorder, which gives rise to an inhomogeneous mechanism of line broadening of optical spectra. This feature is characteristic of all the crystal hosts considered in this study.

### 4.1. Gd_3_Al_2.5_Ga_2.5_O_12_ (GAGG) Host

**Figure 2:**
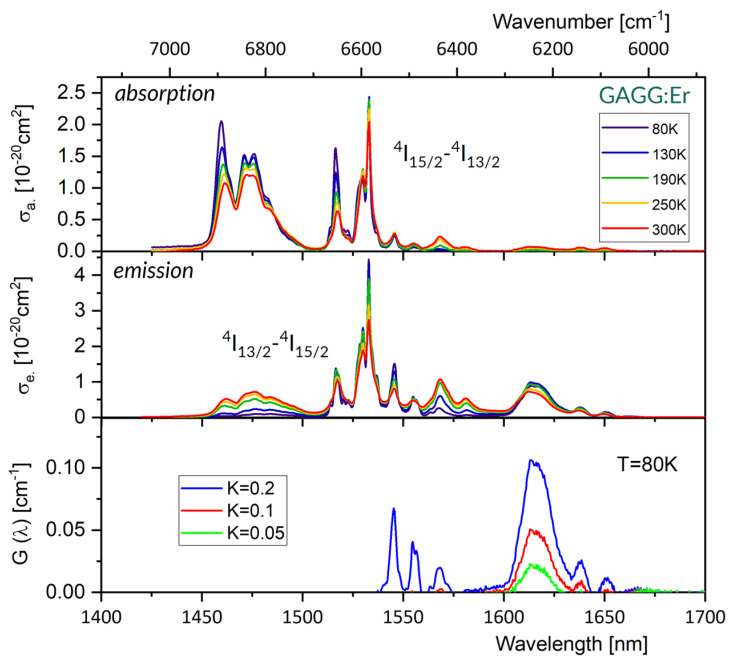
Absorption cross-section spectra (**upper**), emission cross-section spectra (**medium**) at several different temperatures between 80 and 300 K and gain G spectra at 80 K (**bottom**) for a GAGG:Er^3+^ single crystal.

Figure 2 compares absorption spectra (upper) and emission spectra (medium) at several different temperatures between 80 and 300 K for a GAGG:Er single crystal. For clarity, spectra for five temperature points were chosen from ten temperature points measured and are shown in this figure. At 300 K, the higher energy crystal field components of the two multiplets involved cannot be assumed empty. Therefore, the spectra measured are envelopes of 56 spectral lines related to transitions between individual crystal field levels. When the temperature diminishes the populations of a higher-energy crystal field levels of initial multiplets become smaller. As a consequence, the spectra at 80 K show more pronounced structures. Nevertheless, a sample needed to be cooled down to 5 K to disclose well-isolated lines of transitions between individual crystal field levels. With these data, the energies of components of the ^4^I_15/2_ ground state are found from the emission spectra to be (in cm^−1^) 0, 21, 54, 74, 385, 401, 491, and 538, and the energies of the components of the ^4^I_13/2_ state are found from absorption spectra to be (in cm^−1^) 6531, 6547, 6580, 6598, 6608, 6796, 6855. Thus, the energy separations between the two lowest components of the ground state and of the excited state are small, amounting to 21 cm^−1^ and 16 cm^−1^, respectively.

At 80 K, the kT~48 cm^−1^ and the Bolztmann statistics indicate that the population of the higher-energy components of initial multiplets is close to 30% of a total multiplet population, and thereby spectra recorded at 80 K remain still complex. Now, it is worth comparing our results to those found for YAG:Er. The problem of crystal field splitting of multiplets and the spectral features of Er transitions in the YAG host, considered first in year 1964 [46], has been revisited in subsequent works. We now refer to a paper by Ter-Gabrielyan et al. [47], which is the most relevant to our study. In [47], the authors reported that the ^4^I_15/2_ multiplet undergoes splitting by the crystal field into two groups, stretching from 0 to 79 cm^−1^ and from 418 to 573 cm^−1^, respectively, while the ^4^I_13/2_ multiplet undergoes splitting in the range of 6548–6886 cm^−1^. Moreover, the energy separation between the two lowest components of the ground state amounts to 21 cm^−1^.

Accordingly, thus far, the dissimilarity of our results with those reported for YAG:Er is rather small. However, in the ordered crystal structure of YAG, the bandwidths of transitions between crystal field levels at 77 K are around ~0.5 nm, and the width of strongest absorption line at ~1532.3 nm is found to be even smaller ~0.02–0.05 nm [47]. For comparison, the bandwidth (FWHM) of the Er absorption line at 1532 nm (0-0 line) was inferred from our absorption spectra to be ~1.75 nm at 80 K and ~2.6 nm at 300 K. It can be seen in Figure 2 that both the absorption and emission spectra stretch from about 1450 nm to about 1655 nm. Their overall width depends weakly on temperature, but the distribution of intensities within their spectra is significant. The change of temperature from 300 K to 80 K results in an approximately two-fold increase in absorption intensity at a local maximum near 1461 nm, an almost three-fold increase in absorption intensity near 1516, and a small increase in absorption intensity (about 20%) at the 0-0 line. Unlike the extremely narrow absorption lines of YAG:Er, the bandwidth of the local absorption maxima at 1516 and 1532 nm for GAGG:Er are close to a typical line-narrowed emission spectrum of a pump diode, which has a bandwidth of about 2 nm. The emission spectrum in Figure 2 indicates that a free-running laser operation in GAGG:Er is expected near 1650 nm.

**Figure 3:**
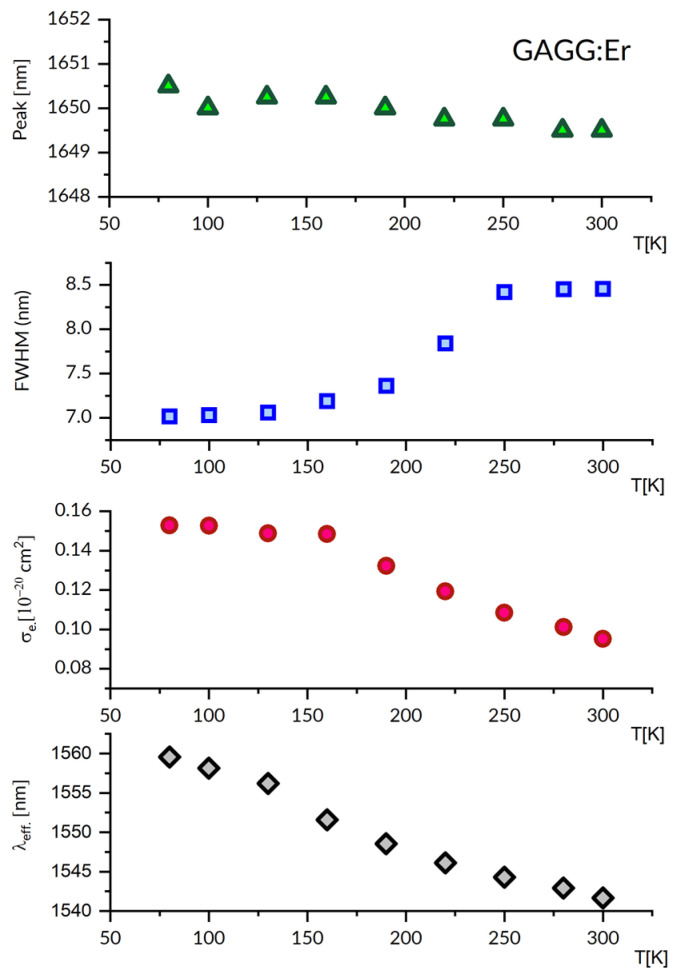
The plots of emission peak wavelength, its FWHM, peak emission cross-section and effective luminescence wavelength, versus temperature in the 80–300 K region for the GAGG:Er^3+^ luminescence band around 1650 nm.

The relevant spectral features of a laser operation at cryogenic temperature follow from Figure 3, which gathers plots of peak emission wavelength, spectral bandwidth, emission cross-section and effective emission wavelength as a function of temperature between 80 K and 300 K. In particular, at 80 K, the peak emission wavelength is nearly the same (within 1 nm), the emission bandwidth is smaller by about 20%, the stimulated emission cross-section is higher by about 60% and the effective emission wavelength is smaller by about 20 nm, respectively, than those at 300 K.

The stimulated emission cross-section values shown deserve comment. They were evaluated using Equation (4), as above. The reliability of radiative lifetime τ is crucial for this evaluation. Therefore, the comparison of τ values determined by two different approaches may point at possible incertitude involved. The (J-O) treatment of a room temperature absorption spectrum of GAGG:Er previously provided τ = 4.81 ms [36]. Inserting a value of oscillator strength P(ED) = 1.26 × 10^−6^ to Equation (7) gives A(ED) = 145 s^−1^ and τ = 5.18 ms (with a contribution of A(MD) = 48 s^−1^). Thus, the consistency of these results is satisfactory.

A graph included in the lowest part of Figure 2 shows a plot of a gain coefficient G(λ) versus wavelength according to Equation (8). This plot indicates that a GAGG:Er laser with a properly chosen dispersive element inserted in a laser cavity would be able to emit radiation tuned between about 1600 nm and 1640 nm. Quite recently, Nemec et al. have published a paper [48] reporting an extensive investigation of a tunable room temperature resonantly pumped GAGG:Er laser, following their previous study [18] on the spectroscopic and laser properties of this crystal as a function of temperature between 80 K and 340 K. Thus, there is an opportunity to check whether our assessments and/or predictions are consistent with results reported in the papers mentioned above. First, in [48], the Judd–Ofelt treatment of the room temperature absorption spectrum of the GAGG:Er sample provided a ^4^I_13/2_ radiative lifetime τ = (5.76 +/− 0.22) ms, which is a value in reasonable agreement with our finding. Second, experimental tuning curves for a resonantly pumped, room temperature GAGG:Er laser between 1609–1622 nm, 1634–1637 nm and 1647–1650 nm observed in [48] are consistent with the predicted tuning region in our Figure 2, as well.

It is worth mentioning that the GAGG:Er laser investigated in [18,48] was pumped with a laser diode emitting at 1461 nm. The energy of pump photons at this wavelength is 6845 cm^−1^. The energy of photons emitted by a laser at 1650 nm is 6061 cm^−1^. Accordingly, the quantum defect (QD), defined as the difference between absorbed and emitted photon energy, amounts to 784 cm^−1^, i.e., QD ~ 11% of the absorbed pump energy that is converted to heat in a laser crystal. This corresponds to an analysis of absorption spectra shown Figure 2 which indicates that components at 1516 and 1532 nm for GAGG:Er may serve as pump bands, thereby diminishing the QD values to 535 cm^−1^ and 466 cm^−1,^ i.e., to 8.1% and 7.6% of absorbed pump energy, respectively.

### 4.2. (Lu_0.3_Gd_0.7_)_2_SiO_5_ (LGSO) Host

**Figure 4:**
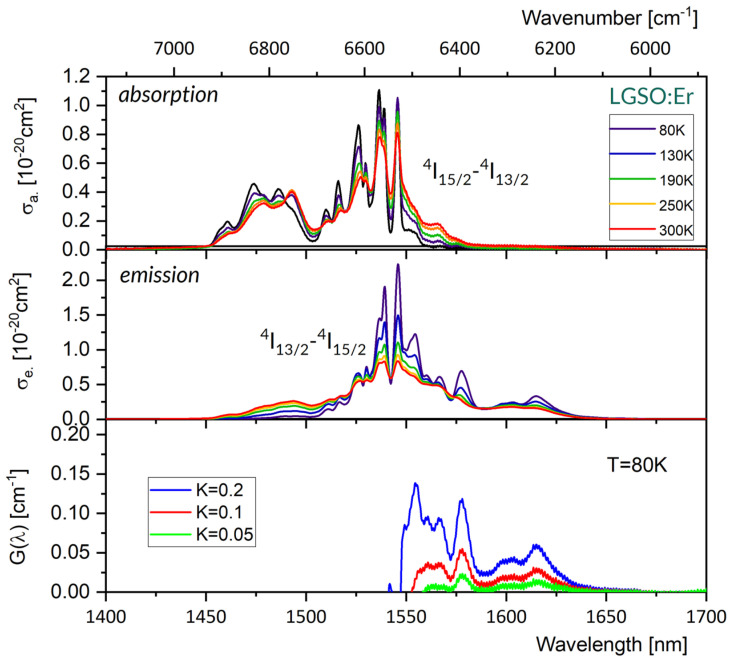
Absorption cross-section spectra (**upper**), emission cross-section spectra (**medium**) at several different temperatures between 80 and 300 K and gain G spectra (**bottom**) at 80 K for a LGSO:Er^3+^ single crystal.

Figure 4 compares absorption cross-section spectra (upper) and emission cross-section spectra (medium) at several different temperatures between 80 and 300 K for a LGSO:Er single crystal. For clarity, spectra for five temperature points were selected from the ten temperature points measured and are shown in this figure.

**Figure 5:**
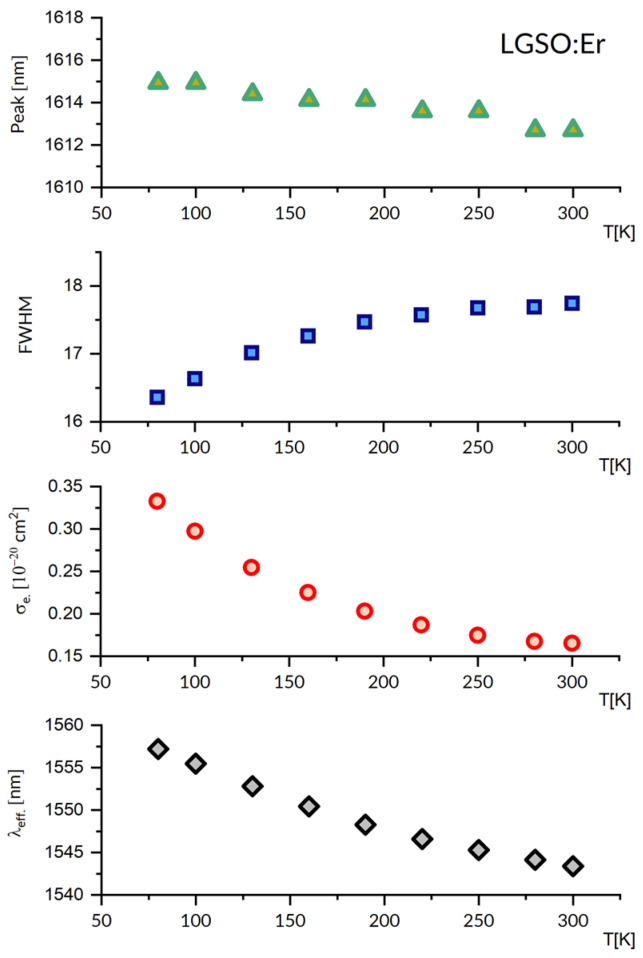
The plots of emission peak wavelength, its FWHM, peak emission cross-section and effective luminescence wavelength, versus temperature in the 80–300 K region for the LGSO:Er^3+^ luminescence band around 1615 nm.

It follows from these plots that both the absorption and emission spectra stretch from about 1450 nm to about 1645 nm, and their overall width depends weakly on the temperature. A change in temperature from 300 K to 80 K results in a redistribution of local maxima within a broad band, contributing to spectra between 1450 and 1500 nm and a slight increase in the intensity of much narrower absorption peaks at 1515, 1526, 1536, 1539 and 1546 nm. The emission spectrum in Figure 4 indicates that a free-running laser operation in LGSO:Er is expected near 1615 nm. The relevant spectral features of a laser operation at cryogenic temperature follow in Figure 5, which gathers plots of peak emission wavelength, spectral bandwidth, emission cross-section and effective emission wavelength as a function of temperature between 80 K and 300 K. At 80 K, the peak emission wavelength is red-shifted by about 2 nm, the emission bandwidth is smaller by about 15%, the stimulated emission cross-section is higher by about 49%, and the effective emission wavelength is smaller by about 30 nm, respectively, compared to those at 300 K. The value of radiative lifetime τ = 4.54 ms obtained from the J-O treatment [37] was inserted into Equation (4) to obtain the stimulated emission cross-section values shown in Figure 4. It is worth noticing that in the J-O calculation procedure, the host crystal anisotropy has been accounted for. In particular, the mean values of oscillator strengths of absorption spectra recorded for polarized light with an E vector parallel to the crystallographic b axis (E||b), an E vector parallel to the optical X1 axis (E||X1), and an E vector parallel to the optical axis X2 (E||X2) were used as input data for the fitting.

Although the band structure of transitions between the ^4^I_13/2_ and ^4^I_15/2_ multiplets are markedly more rich at 80 K than at 300 K, the sample should be cooled down to 6 K to disclose lines for transitions between crystal field levels. Ten crystal field levels located at 6497, 6508, 6551, 6563, 6598, 6622, 6724, 6787, 6847 and 6866 cm^−1^ were then found for the ^4^I_13/2_ multiplet, and six crystal field levels were located at 0, 23, 58, 116, 160 and 310 cm^−1^ for the ground state [37]. Thus, the energy separation between the two lowest components of the ground and excited states is small, and at 80 K, the spectra recorded remain still complex. Additionally, it follows from these data that the 0-0 line is located at 1539 nm. Examination of the absorption spectra in Figure 4 reveals that lines at 1536, 1539 and 1546 nm are intense and advantageously broad to serve as pump bands. In particular, the QD arising from a difference between the pump wavelength of 1546 nm and the emission wavelength of 1616 nm equals 280 cm^−1,^ i.e., 4% of absorbed pump energy. A graph included in the lowest part of Figure 4 shows a plot of the gain coefficient G(λ) versus wavelength, according to Equation (8). This plot indicates that a LGSO:Er laser with a properly chosen dispersive element inserted in a laser cavity would be able to emit radiation tuned between about 1550 nm and 1615 nm.

To our knowledge, laser operation in LGSO:Er at cryogenic temperature has not been reported yet. Nevertheless, some spectroscopic results reported for isostructural Y_2_SiO_5_ (YSO) and Lu_2_SiO_5_ (LSO)-ordered hosts and LGSO co-doped with Er and Yb are relevant to our study, and are therefore worth commenting on. In [49], which is devoted to Y_2_SiO_5_ doped with Er and co-doped with Yb, the authors found that the ^4^I_13/2_↔^4^I_15/2_ spectra recorded at 10 K consist of 14 lines in absorption and 16 lines in emission, which is in agreement with the number of crystal field components of excited and ground states for two different Er^3+^ sites. The ^4^I_13/2_ radiative lifetime τ = 5.60 ms was then determined based on the J-O treatment of absorption spectra. On the other hand Y.C. Ding et al. [50] inferred from absorption spectra recorded at 10 K that the ^4^I_15/2_ ground state of Er^3+^ in the Lu_2_SiO_5_ (LSO) host has ten crystal field components, and the overall multiplet splitting amounts to 197 cm^−1^ [50]. In their study, the authors also observed a laser operation at 1596 nm in LSO:Er, with an output power of 610 mW upon resonant optical pumping. In their earlier paper, Y.C. Ding et al. reported the ^4^I_13/2_ radiative lifetime τ = 4.58 ms, itself derived from the J-O treatment of room temperature absorption spectra of Er^3+^ in Lu_2_SiO_5_ (LSO) [51,52]. Lin Han et al. investigated up-conversion phenomena in LSO and LGSO co-doped with Er^3+^ and Yb^3+^ at temperatures in the region of 260 K–470 K. They determined radiative lifetime values of 5.15 ms and 4.43 ms for the ^4^I_13/2_ level for LSO:Er,Yb and LGSO:Er,Yb, respectively, and a peak value of a room temperature emission cross-section for Er^3+^ in LGSO:Er,Yb of 0.994 × 10^−20^ cm^2^ at 1543 nm. For comparison, a corresponding value of 0.81 × 10^−20^ cm^2^ can be inferred from our Figure 4.

### 4.3. LiNbO_3_ (LNO) Host

**Figure 6:**
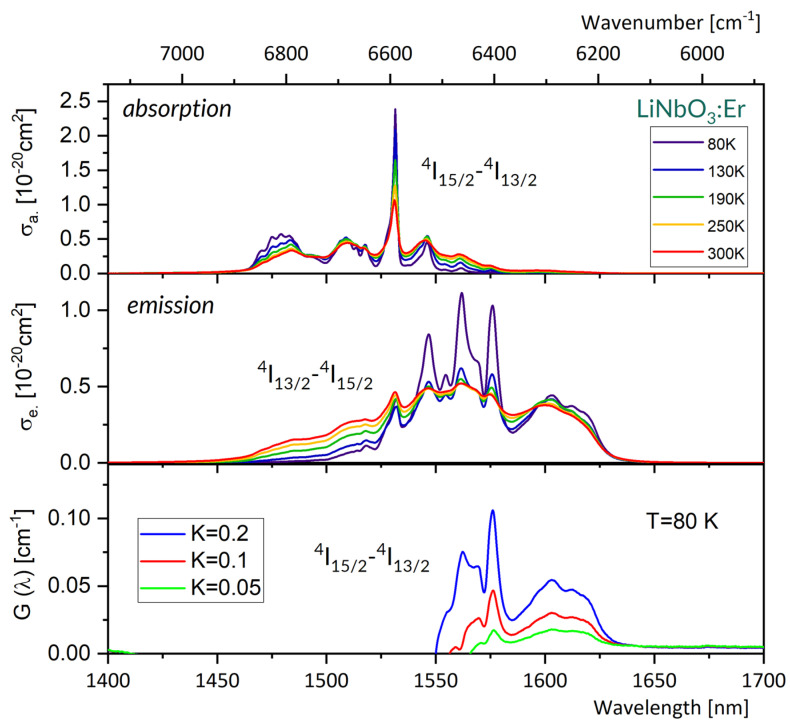
Absorption cross-section spectra (**upper**), emission cross-section spectra (**medium**) at several different temperatures between 80 and 300 K and gain G spectra at 80 K (**bottom**) for a LNO:Er^3+^ single crystal.

Figure 6 compares absorption spectra (upper) and emission spectra (medium) at several different temperatures between 80 and 300 K for a LNO:Er single crystal. For clarity, spectra for five temperature points were selected from ten temperature points measured and are shown in this figure. It follows from these plots that both the absorption and emission spectra stretch from about 1450 nm to about 1645 nm, and their overall width depends weakly on the temperature. A change in temperature from 300 K to 80 K results in a redistribution of local maxima within a broad band, contributing to spectra between 1450 and 1500 nm and a slight increase in the intensity of much narrower absorption peaks at about 1510, 1531, and 1546 nm.

The crystal field splitting of multiplets involved in the ^4^I_15/2_–^4^I_13/2_ transition has been determined from low temperature spectra of LNO:Er and reported in several published papers. V.T. Gabrielyan et al. [53] reported a value 6524 cm^−1^ for the lowest energy component of the ^4^I_13/2_ multiplet, and overall ground state splitting of 414 cm^−1^ (at 77 K). G. Dominiak-Dzik et al. [38] located the lowest energy component of the ^4^I_13/2_ multiplet at 6531 cm^−1^, and reported overall splitting amounting to 443 cm^−1^ for the ^4^I_15/2_ ground state at 5 K. According to V.G. Babajanyan [54] the lowest energy component of the ^4^I_13/2_ is located at 6529 cm^−1^, and the overall ^4^I_15/2_ splitting is 415 cm^−1^. J.B. Gruber et al. [55] thoroughly considered the crystal field splitting of Er^3+^ multiplets in LNO crystal by comparing the calculated splitting values to experimental data obtained from absorption spectra at 8 K and emission spectra at 8 K and 80 K. They located the lowest energy component of the ^4^I_13/2_ multiplet at 6531.3 cm^−1^ (from absorption spectra at 8 K), at 6531.4 cm^−1^ (from emission spectra at 8 K), and at 6532 cm^−1^ (from emission spectra at 80 K). The findings mentioned above are consistent and indicate that the prominent line located at about 1531 nm in the absorption spectra in Figure 6 is the 0-0 line.

Unlike the 0-0 lines observed in GGAG:Er and LGSO:Er, the width of the 0-0 line in LNO:Er is small. It amounts to about 2.3 nm at 300 K; next, it decreases monotonously with decreasing temperature, reaching a value of about 1 nm at 80 K. The reason for this is that the energy difference between the lowest-energy crystal field components and the next-highest energy components in the ^4^I_15/2_ and the ^4^I_13/2_ multiplets is quite large, amounting to about 60 cm^−1^ and 81 cm^−1^, respectively [38,54,55]. As a consequence, the populations of the higher energy components are small, thereby preventing the contribution of overlapping components in spectra at 80 K.

**Figure 7:**
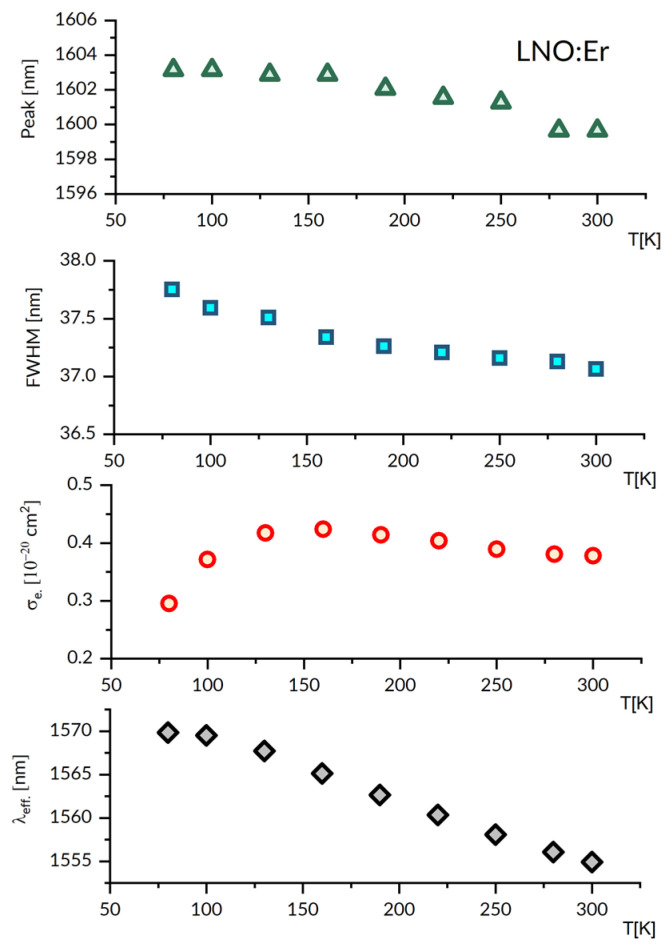
The plots of emission peak wavelength, its FWHM, peak emission cross-section and effective luminescence wavelength, versus temperature in the 80–300K region for the LNO:Er^3+^ luminescence band around 1603 nm.

The emission spectrum in Figure 6 indicates that a free-running laser operation in LNO:Er is expected to be near 1603 nm. The relevant spectral features of a laser operation at a cryogenic temperature follow from Figure 7, which gathers plots of peak emission wavelength, spectral bandwidth, emission cross-section and effective emission wavelength versus temperature between 80 K and 300 K.

These plots indicate that at 80 K, the peak emission wavelength is red-shifted by about 2 nm, the emission bandwidth is higher by about 3%, the stimulated emission cross-section is lower by about 22%, and the effective emission wavelength is higher by about 14 nm, as compared to those at 300 K, respectively. The reliability of the stimulated emission cross-section values plotted in Figure 7 deserves some comment. It follows from Equation (4) that a governing factor is radiative lifetime value τ for the transition involved. Nunez et al. [56] reported τ = 2.72 ms, Amin et al. [57] reported τ = 2.7 +/− 0.4 ms, Ahua Li et al. [58] reported τ = 2.19 ms, and Yannan Qian at al [59] reported τ = 2.46 and 5.77 ms for Er^3+^ concentrations of 0.5 mol% and 3 mol%, respectively. All the lifetime values mentioned above have been obtained from the J-O treatment of room temperature absorption spectra. For comparison, we obtained τ = 2.90 ms with P(ED)=2.06 × 10^−6^ inserted into Equation (7).

The lowest graph in Figure 6 shows a plot of gain coefficient G(λ) versus wavelength according to Equation (8). This plot indicates that a LNO:Er laser with a properly chosen dispersive element inserted into a laser cavity would be able to emit radiation tuned between about 1550 nm and 1640 nm for K ≤ 0.2, and possibly also at a peak located at 1546 nm for K > 0.2. When pumping at 1531 nm, the energy of pump photons is 6532 cm^−1^. The energy of photons emitted by a laser at 1603 nm or at 1546 nm is 6238 cm^−1^ and 6468 cm^−1^, respectively. Accordingly, a respective quantum defect (QD), defined as a difference between absorbed and emitted photon energy, amounts to 294 cm^−1^ or 64 cm^−1^, i.e., QD ~ 5% or 1% of the absorbed pump energy that is converted to heat in a laser crystal.

For comparison, the smallest values of the quantum defect (QD) are about 7.6%, 4% and 1% for GAGG:Er, LGSO:Er and LNO:Er, respectively. These findings corroborate the supposition that the adverse heating related to quantum defect for erbium-doped laser crystals induced by resonant on-line optical pumping is very small. However, the phenomenon of heat generation in laser media deserves some more attention. In principle, in addition to QD, it may result from the contribution of nonradiative transitions to the depletion of the upper laser level. This energy loss is not crucial for continuous laser operation (CW regime) because during laser action, the rate of stimulated emission is markedly higher than that of combined spontaneous emission and nonradiative decay. However, it may cause more adverse effect during the pulsed Q-Switch operation, especially for low duty factors. The ^4^I_13/2_ upper laser multiplet is the lowest energy-excited level of the Er^3+^ ion; therefore, nonradiative decay due to a cross-relaxation mechanism is excluded, but the multiphonon relaxation process may contribute. Usually, the rate of nonradiative transitions for an excited level is obtained by subtracting the inverse of an experimental luminescence’s lifetime from the inverse of its radiative lifetime. Unfortunately, experimental lifetimes recorded from the luminescence decay curves for the lowest energy-excited levels of trivalent rare earth ions are not reliable due to the adverse phenomenon of self-absorption. The problem is that the researcher cannot find any evidence that their efforts devoted to obtaining actual data have been successful. To overcome this difficulty, we made use of a phenomenological approach known as “the energy gap law”, which relies on data obtained for several excited levels differing in energy. In this way, rates of multiphonon relaxation of the ^4^I_13/2_ level of 5 s^−1^ [48], 6 s^−1^ [37] and 6 s^−1^ [38] were obtained for GAGG:Er, LGSO:Er and LNO:Er, respectively, assuming the respective cut-off phonon energies given in Table 1. Accordingly, we conclude that the heat generation induced in the system studied by a nonradiative relaxation of the upper laser level is negligible.

In the GAGG host, a structural disorder occurs in the second coordination sphere of Er^3+^ ions. Therefore, it weakly affects the crystal field splitting specific to garnets, and induces rather small inhomogeneous line broadening of Er^3+^ transitions. In the LGSO host, the Er^3+^ ions are located in the Gd^3+^ and Lu^3+^ sites and are exposed to a disorder in their first coordination sphere. As a consequence, their crystal field splitting is affected markedly, and induced line broadening is significant. In the LNO host, the Er^3+^ ions are exposed to a crystal field distorted strongly by a disparity in the ionic radii of substituted ions combined with a disorder resulting from a charge compensation problem. The proposed interpretation accounts well for thermally induced changes in spectroscopic factors.

## 5. Conclusions

It was found that at 80 K, the spectral width of the emission bands at the predicted wavelengths of free-running laser operation amount to 7 nm, 16.5 nm and 37.8 nm for GAGG:Er, LGSO:Er and LNO:Er, respectively. Significant differences in the bandwidths given above were attributed to dissimilarities in the strength and symmetry of the crystal field in Er^3+^ sites combined with different origins of line broadening effect. A change in temperature from 300 K to 80 K results in a 20% decrease in the bandwidth and an increase of 60% in the peak value of the stimulated emission cross-section for GAGG:Er, and a 15% decrease in bandwidth and increase by 49% in the peak value of the stimulated emission cross-section for LGSO:Er. The peak value of the stimulated emission cross-section decreases by 22%, and the bandwidth is unaffected by the same change of temperature for LNO:Er. This investigation provides results that are relevant to lasing ability of systems studied at cryogenic temperatures. In particular, the tuning range of the output laser wavelength was determined to be 40 nm, 63 nm and 53 nm for GAGG:Er, LGSO:Er and LNO:Er, respectively. Inhomogeneous broadening of absorption bands makes it possible to advantageously reduce the quantum defect (QD) value to about 7.6%, 4% and 1% for GAGG:Er, LGSO,Er and LNO:Er, respectively.

## Figures and Tables

**Table 1:** Selected physicochemical properties of the GAGG, LGSO and LNO single crystals.

	GAGG	LGSO	LNO
Crystal structure	Ia-3d, cubic a = 12.22 Å V = 1826.27 Å^3^, Z = 8	P1 21/c1, monoclinic a = 8.99 Å, b = 6.71 Å c = 6.59 Å, β = 104.18^o^ V = 385.36 Å^3^, Z = 4	R3c, trigonal a = 5.15 Å, b = 5.15 Å, c = 5.49 Å, α = 62.057°, β = 62.057°, γ = 60°, Z = 6
Density [g/cm^3^]	6.63	6.65	4.64
Thermal conductivity [W/mK]	6.70	3.70	4.60
Refractive index	1.96	1.85	2.30
Phonon energy [cm^−1^]	808	900	879
Energy gap [eV]	5.90	5.95	3.77

## Data Availability

The data presented in this study are available on request from the corresponding author.
